# Image Processing Hardware Acceleration—A Review of Operations Involved and Current Hardware Approaches

**DOI:** 10.3390/jimaging10120298

**Published:** 2024-11-21

**Authors:** Costin-Emanuel Vasile, Andrei-Alexandru Ulmămei, Călin Bîră

**Affiliations:** Department of Electronic Devices, Circuits and Architectures, National University of Science and Technology Politehnica Bucharest, 060042 Bucharest, Romania; calin.bira@upb.ro

**Keywords:** image processing, neural networks, hardware accelerators, FPGA

## Abstract

This review provides an in-depth analysis of current hardware acceleration approaches for image processing and neural network inference, focusing on key operations involved in these applications and the hardware platforms used to deploy them. We examine various solutions, including traditional CPU–GPU systems, custom ASIC designs, and FPGA implementations, while also considering emerging low-power, resource-constrained devices.

## 1. Introduction

Image processing, especially in AI and computer vision, requires faster and more efficient data handling, making hardware acceleration (with dedicated hardware like Application-Specific Integrated Circuits-ASICs, Graphics Processing Units-GPUs) essential, as general-purpose CPUs (GP-CPUs) face performance limitations in real-time, high-resolution processing due to their lack of parallelism and lack of focus on energy efficiency.

Designing hardware to accelerate image processing and neural network (NN) workloads requires analyzing commonly used operations. Our work aims to provide a comprehensive analysis of these operations and hardware architectures, effectively helping developers to select the appropriate hardware type or guiding hardware designers in creating more efficient and higher-performing image processors. The remainder of this paper is structured as follows: Section 2 reviews the common operations used in machine learning, offering insights into the features future image processors should support. Section 3 provides an overview of current hardware platform options, ranging from traditional CPU–GPU systems to Field-Programmable Gate Arrays (FPGAs) and more resource-constrained hardware, such as microcontrollers. Section 4, Section 5 and Section 6 present concluding remarks based on our analysis, highlighting research gaps and challenges in hardware design for image processing.

## 2. Operations Used in Machine Learning Applications

This section provides a comprehensive overview of the foundational operations that are most commonly employed in machine learning applications. The focus is particularly directed towards techniques and methodologies relevant to image processing.

### 2.1. Operations Used in Convolutional Neural Networks

The main machine learning network architecture used in image processing is the convolutional neural network (CNN). This section will provide an in-depth analysis of the operations in the CNN architecture (Table 1).

#### 2.1.1. Convolution

Convolution is a fundamental operation in convolutional neural networks, which are widely used in image processing and computer vision tasks. The convolution operation enables CNNs to extract spatial features from input data, such as edges, textures, and patterns, which are essential for tasks like image classification, object detection, and segmentation [8,9,10].

The convolution operation fundamentally involves sliding a filter (or kernel) over an input image to produce a feature map. The filter is a small matrix (typically of size 3 × 3 or 5 × 5) of learnable weights applied to a portion of the input image. The convolution operation can be mathematically expressed as

(1) $$y\left(i,j\right)=\sum_{m=-k}^{k}\sum_{n=-k}^{k}x\left(i+m,j+n\right)\cdot w\left(m,n\right)$$

where y(i,j) is the value of the feature map at position (i,j), x(i+m,j+n) is the value of the input image at position (i+m,j+n), w(m,n) is the filter weight at position (m,n), and *k* is the half-width of the filter.

In addition to basic convolution, CNNs often employ strided convolution, where the filter moves by more than one pixel at a time. This reduces the spatial dimensions of the output feature map and can be particularly useful for downsampling [11]. The formula for strided convolution is

(2) $$y\left(i,j\right)=\sum_{m=-k}^{k}\sum_{n=-k}^{k}x\left(i+s\cdot m,j+s\cdot n\right)\cdot w\left(m,n\right)$$

where *s* is the stride, representing the number of pixels the filter moves forward after each operation.

Padding is often added to the input image to control the size of the output feature map. Zero-padding is the most common technique, adding zeros around the image’s borders. Padding allows the filter to be applied to the edges of the image, preserving the spatial dimensions of the input [2]. The effect of padding can be described by the formula:

(3) $$Outputsize=\frac{InputSize-FilterSize+2\cdot Padding}{Stride}+1$$

where InputSize is the dimension of the input image, FilterSize is the dimension of the filter, and Padding is the number of pixels added to the input image.

Other types of convolution are prominent in the literature, as follows:

- Depthwise convolution
- Pointwise convolution
- Depthwise separable convolution

##### Depthwise Convolution

In depthwise convolution, instead of applying a single convolutional filter across all input channels, a separate filter is applied to each input channel independently [12]. For an input $x_{c}$ with *C* channels and a filter $w_{c}$ applied to the *c*-th channel, the operation is

(4) $$y_{c}\left(i,j\right)=\sum_{p=0}^{m-1}\sum_{q=0}^{n-1}w_{c}\left(p,q\right)\cdot x_{c}\left(i+p,j+q\right)$$

where $y_{c}\left(i,j\right)$ is the output of the *c*-th channel, and $w_{c}\left(p,q\right)$ is the filter applied to the *c*-th channel.

##### Pointwise Convolution

Pointwise convolution uses a 1 × 1 filter across the input channels, which essentially mixes the channels at each spatial location [13]. The operation for a pointwise convolution can be expressed as

(5) $$y\left(i,j\right)=\sum_{c=1}^{C}w_{c}\cdot x_{c}\left(i,j\right)$$

where y(i,j) is the output at location (i,j), $w_{c}$ is the weight for the *c*-th channel, and $x_{c}\left(i,j\right)$ is the input value from the *c*-th channel.

##### Depthwise Separable Convolution

Depthwise separable convolution is a combination of depthwise convolution followed by pointwise convolution. The depthwise part applies separate filters to each input channel, and the pointwise part combines these filtered outputs across channels. The complete operation is

(6) $$y_{c}\left(i,j\right)=\sum_{k=1}^{C}w_{k}\cdot \left(\sum_{p=0}^{m-1}\sum_{q=0}^{n-1}w_{c,k}\left(p,q\right)\cdot x_{k}\left(i+p,j+q\right)\right)$$

where $y_{c}\left(i,j\right)$ is the output of the *c*-th channel, $w_{k}$ is the pointwise convolution filter, and $w_{c,k}$ is the depthwise convolution filter for the *k*-th channel.

#### 2.1.2. Pooling

Pooling is a critical operation in convolutional neural networks (CNNs), reducing the spatial dimensions of feature maps, while retaining the most essential information. By downsampling the input, pooling operations help to decrease the computational load, reduce the number of parameters, and control overfitting, all of which are crucial for the efficiency and effectiveness of deep learning models. This section explores the various types of pooling operations, including max pooling, average pooling, global pooling, and more specialized methods like L2 pooling and fractional pooling [14,15].

##### Max Pooling

Max pooling operates by sliding a fixed-size window (usually 2 × 2, 3 × 3) over the input feature map (usually the output of a convolutional layer) and selecting the maximum value within the window. This operation is repeated across the entire feature map, effectively downsampling the input while preserving the most prominent features The formula for max pooling can be expressed as

(7) $$P_{max}\left(i,j\right)=\max_{m,n}\left(f\left(x_{i+m,j+n}\right)\right)$$

where $P_{max}\left(i,j\right)$ is the pooled output at position (i,j), $f(x_{i+m,j+n})$ represents the input values within the pooling window, and m,n iterate over the dimensions of the pooling window.

##### Average Pooling

Average pooling operates similarly to max pooling, but instead of selecting the maximum value, it computes the average of all the values within the pooling window. The formula for average pooling is given by

(8) $$P_{avg}\left(i,j\right)=\frac{1}{\left|W\right|}\sum_{m,n}f\left(x_{i+m,j+n}\right)$$

where $P_{avg}\left(i,j\right)$ is the averaged output at position (i,j), $f(x_{i+m,j+n})$ represents the input values within the pooling window, and |W| is the number of elements in the pooling window.

##### Global Pooling

Global pooling is a particular type of pooling operation applied over the entire feature map rather than a window. It is commonly used at the end of the later layers of a CNN to convert feature maps into a single value per feature map, often being used in fully connected layers for classification applications. There are two main types of global pooling: global max pooling and global average pooling.

Global max pooling selects the maximum value across the entire feature map, as follows:

(9) $$P_{global\_max}=\max_{i,j}\left(f\left(x_{i,j}\right)\right)$$

Global average pooling computes the average value across the entire feature map:

(10) $$P_{global\_avg}=\frac{1}{N}\sum_{i,j}f\left(x_{i,j}\right)$$

##### L2 Pooling

L2 pooling is a less commonly used pooling method that involves computing the L2 norm (Euclidean norm) within the pooling window. This method smooths the feature map by penalizing large values [16] and is defined as

(11) $$P_{L2}\left(i,j\right)=\sqrt{\sum_{m,n}{\left(f\left(x_{i+m,j+n}\right)\right)}^{2}}$$

L2 pooling is useful in scenarios where the network requires a regularized feature map representation, particularly in networks where activation values might be too large.

##### Fractional Pooling

Fractional pooling is a pooling technique used to downsample feature maps more flexibly than traditional pooling methods, which typically involve integer strides. Fractional pooling allows non-integer strides, providing finer control over the downsampling process.

Fractional pooling can be achieved through interpolated pooling and stochastic fractional pooling.

Fractional pooling can be realized using interpolation methods, where the stride is fractional and the pooling windows overlap slightly. The pooling output is computed as a weighted sum of overlapping regions [17], described by the formula:

(12) $$P_{f}\left(i,j\right)=\left(1-\alpha \right)\cdot P_{S_{1}}\left(i,j\right)+\alpha P_{S_{2}}\left(i,j\right)$$

where $S_{f}$ is a fractional number between $S_{1}$ and $S_{2}$, α is the interpolation factor determined by the fractional part of $S_{f}$, and $P_{S_{1}}$ and $P_{S_{2}}$ are the pooling outputs using strides $S_{1}$ and $S_{2}$, respectively.

Another method of implementing fractional pooling is stochastic fraction pooling, where the stride is chosen probabilistically based on the desired fractional value. This method does not rely on interpolation; instead, it randomly selects between two adjacent pooling regions.

##### Convolution and Pooling in Hardware Acceleration

Convolution and pooling operations are critical components in convolutional neural networks (CNNs) for feature extraction and dimensionality reduction. Given their computational intensity, especially in deep neural networks with large input sizes, multiple hardware platforms and techniques have been developed to accelerate these operations, particularly for real-time applications.

- GPUs (graphics processing units): GPUs are highly parallel processing units ideal for large-scale matrix operations such as convolution, due to their ability to perform multiple convolutions concurrently. Techniques like memory access optimization (efficiently utilizing the GPU memory hierarchy), parallelism, and kernel optimization (including loop unrolling, tiling, and reducing thread divergence) improve convolution performance significantly [18]. Pooling operations on GPUs are similarly optimized through parallelization, with independent pooling operations performed simultaneously and memory access optimization achieved via coalesced memory access, consolidating contiguous memory addresses into a single transaction [19].
- TPUs (tensor processing units): TPUs are specialized accelerators developed by Google specifically for deep learning tasks, and they excel in operations central to convolution, like matrix multiplications. TPUs use systolic arrays to pipeline matrix multiplications, which reduces latency and improves convolution efficiency [20]. Although pooling does not involve matrix multiplications, TPUs’ optimized data flow architecture minimizes the data movement during pooling, leveraging the same efficient systolic array configuration.
- FPGAs (field-programmable gate arrays): FPGAs allow for custom hardware configurations tailored to convolution operations, making them particularly useful in low-power, real-time applications like embedded systems. Convolution in FPGAs is accelerated through techniques like pipelining (executing multiple convolution stages in parallel to maintain a continuous data flow), loop unrolling, and dataflow optimization, which minimizes bottlenecks by managing the data movement between memory and processing elements efficiently [21]. For pooling, FPGAs can be configured with custom logic to directly implement and optimize specific pooling operations.
- ASICs (application-specific integrated circuits): ASICs offer the highest level of optimization for specific applications, embedding the convolution and pooling operations directly into hardware for peak efficiency, although they lack flexibility, as they are purpose-built for defined tasks [22]. This direct implementation using ASICs achieves the maximum performance but is only practical in high-volume applications where reconfigurability is not required.
- CPUs (central processing units): While not as specialized as other accelerators, CPUs can still optimize convolution operations using SIMD (single instruction, multiple data) instructions, cache optimization, and multi-core processing [23]. For pooling, CPUs leverage similar parallel and memory access optimization strategies to enhance performance within their architectural limits.

By leveraging the strengths of each platform, hardware acceleration of convolution and pooling can be tailored to fit various application requirements, from high-performance data centers to resource-constrained edge devices.

#### 2.1.3. Activation Functions

Activation functions are components of CNNs that introduce non-linearity into the model, allowing it to learn complex patterns and relationships within the data. Without activation functions, a neural network would behave as a simple linear model, severely limiting its capacity to model intricate patterns. Various activation functions are employed in CNNs, each with their specific properties and advantages. This section explores the most widely used activation functions, their mathematical formulations, and how they are optimized across different hardware platforms.

One concept to mention in this section is the vanishing gradient problem, which is discussed for each activation function. The vanishing gradient problem occurs during the training of deep neural networks when the gradients of the loss function become very small with respect to the model’s parameters. As a result, updates to the weights during backpropagation become insignificant, causing the learning process to slow down or even stop. This problem is particularly pronounced in networks with many layers and it can hinder the training of deep models, especially when using activation functions like sigmoid or tanh, which squash inputs into a small range, leading to gradients that are close to zero [24].

##### Sigmoid Activation Function

The sigmoid function is one of the earliest activation functions used in neural networks. It maps the input values to a range between 0 and 1, making it particularly useful for binary classification tasks [25]

(13) $$\sigma \left(x\right)=\frac{1}{1+e^{-x}}$$

where *x* is the output from the last neuron.

One of the notable properties of the sigmoid function is its smooth gradient, but it suffers from the vanishing gradient problem, especially in deep networks.

##### Tanh Activation Function

The tanh function is a scaled version of the sigmoid function, mapping input values to a range between −1 and 1. It is often preferred over sigmoid because it centers data around zero, leading to a faster convergence during training [25].

(14) $$tanh\left(x\right)=\frac{e^{x}-e^{-x}}{e^{x}+e^{-x}}$$

where *x* is the output of the last neuron.

This function still suffers from a vanishing gradient, but less so than the sigmoid activation function.

##### ReLU (Rectified Linear Unit) Activation Function

The ReLU is the most widely used activation function in CNNs, due to its simplicity and effectiveness. It introduces non-linearity by setting all negative inputs to zero, which allows the network to learn complex patterns more efficiently [1].

(15) ReLU(x)=max(0,x)

This activation function is less prone to the vanishing gradient problem, but it can lead to the “dying ReLU” problem, where neurons stop learning if they consistently output 0.

##### Leaky ReLU Activation Function

A leaky ReLU is a variant of ReLU designed to mitigate the “dying ReLU” problem. Instead of setting negative inputs to zero, it allows a small, non-zero gradient for negative inputs [26].

(16) $$\mathrm{Leaky}\;\mathrm{ReLU}\left(x\right)=\left\{\begin{array}{cc} x, & \mathrm{if}\;x>0\\ \alpha x, & \mathrm{if}\;x\leq 0 \end{array}\right.$$

where α is a small constant, typically around 0.01.

The leaky ReLU’s properties include allowing a small gradient when *x* is negative, helping to keep the neurons alive and helping solve the vanishing gradient problem more effectively than standard ReLU [26].

One variant of leaky ReLU is *parametric ReLU*, which, instead, utilizes a fixed parameter α, whose value can be learned during the training process.

##### Exponential Linear Unit (ELU) Activation Function

An ELU is similar to ReLU but tends to converge faster and produce more accurate results by smoothing the output for negative values.

(17) $$ELU\left(x\right)=\left\{\begin{array}{cc} x, & \mathrm{if}\;x>0\\ \alpha \cdot (e^{x}-1), & \mathrm{if}\;x\leq 0 \end{array}\right.$$

where α is a hyperparameter that controls the value at which an *ELU* saturates for negative net inputs.

##### Swish Activation Function

Swish is a newer activation function developed by Google researchers. It has been shown to perform better than ReLU in some deep networks [27].

(18) $$Swish\left(x\right)=\frac{x}{1+e^{-x}}=x\cdot \sigma \left(x\right)$$

where σ(x) is the sigmoid activation function.

Swish has a smooth curve and does not suffer from the dying ReLU problem, making it a strong candidate for deep networks, particularly in architectures where the gradient flow is critical, such as in very deep networks or networks with skip connections [28].

##### Mish Activation Function

Mish is another activation function that has gained attention for its performance improvements in deep learning tasks. It is defined as follows:

(19) $$Mish\left(x\right)=x\cdot tanh(ln\left(1+e^{x}\right))$$

Mish combines the benefits of a smooth, non-monotonic activation and unbounded output, leading to better performance in some cases compared to ReLU and its variants.

##### Activation Functions in Hardware Acceleration

Activation functions, while computationally simple, are called billions of times during the training of large models, making their efficient implementation crucial for the overall speed of deep learning systems. Custom hardware accelerators such as GPUs, TPUs, and ASICs are designed to handle the massive parallelism required for deep learning, but the specific choice of activation function can influence the design and optimization of these hardware solutions. ReLU and its variants, like leaky ReLU and PReLU, involve straightforward mathematical operations—maximum, multiplication, and addition—which are well-suited to parallel execution on GPUs and TPUs. Custom hardware can further optimize these operations by implementing dedicated circuits that execute these functions with minimal latency, enabling faster inference and training.

Swish and Mish, while more complex than ReLU, can also benefit from hardware acceleration. The computation of the sigmoid function in Swish and the exponential and logarithmic operations in Mish can be optimized through specialized hardware units that perform these operations more efficiently than general-purpose processors. Furthermore, by leveraging low-precision arithmetic, which is increasingly used in custom hardware to speed up computation and reduce power consumption, these functions can be computed more rapidly, without significant loss of accuracy.

For real-time applications or on-device inference, such as in mobile or embedded systems, the choice of activation function and its hardware implementation can have a significant impact on performance. Custom hardware that accelerates these operations can make the difference between a feasible real-time application and one that is too slow to be practical.

#### 2.1.4. Fully Connected Layers

Fully connected layers (FC layers) are a fundamental component in many deep learning architectures, particularly in convolutional neural networks (CNNs). Unlike convolutional layers, which preserve spatial relationships by learning the spatial hierarchies of features, fully connected layers “flatten” the input and connect every neuron in one layer to every neuron in the next. These layers are typically used at the end of CNNs to perform tasks such as classification, where the goal is to assign a label to the input image based on the features extracted by the preceding convolutional and pooling layers [29].

A fully connected layer takes an input vector and applies a linear transformation, followed by a non-linear activation function. The linear transformation is defined as

(20) y=Wx+b

where *x* is the input vector (flattened feature map from the previous layer), *W* is the weight matrix, *b* is the bias vector, and *y* is the output vector. Each element $y_{j}$ of the output vector is computed as

(21) $$y_{j}=\sum_{i=1}^{n}W_{ji}x_{i}+b_{j}$$

where *n* is the number of input neurons, $W_{ji}$ represents the weight connecting the *i*-th input neuron to the *j*-th output neuron, $x_{i}$ is the *i*-th input value, $b_{j}$ is the bias for the *j*-th output neuron.

In CNNs, fully connected layers combine the features extracted by convolutional layers to make final predictions. After the convolutional layers have detected various features across different spatial hierarchies, the fully connected layers integrate this information to form the final decision boundaries. This is particularly important in classification tasks, where the last fully connected layer typically outputs the logits (unnormalized probabilities) that correspond to the different classes.

Some common variants of fully connected layers exist:

- Dense Layers: The standard fully connected layer is described above, where each neuron is connected to every neuron in the previous layer.
- Dropout Layers: Often applied to fully connected layers, dropout randomly sets a fraction of the neurons to zero during training, which prevents overfitting by encouraging the network to learn redundant representations.
- Batch Normalization: Sometimes applied after fully connected layers, batch normalization normalizes the output of the layer, which can accelerate training and improve model performance.

##### Fully Connected Layers in Hardware Acceleration

The FC layers can be efficiently optimized using hardware acceleration, as follows:

- GPUs are highly effective at accelerating fully connected layers, due to their ability to perform massive parallel matrix multiplications.
- TPUs use systolic arrays, a highly efficient architecture for performing large-scale matrix multiplications, which is the core operation in fully connected layers. This architecture allows TPUs to process fully connected layers with high throughput and low latency. TPUs often employ quantized operations, using lower precision (e.g., 8-bit integers) to accelerate the computation of fully connected layers, while maintaining sufficient accuracy [20].
- FPGAs can be programmed to execute fully connected layers with custom logic tailored for matrix multiplications. This allows for highly efficient data paths and parallel processing of matrix operations, reducing latency and power consumption. FPGAs can be designed to process different parts of the matrix multiplication concurrently (pipeline architecture), which optimizes the throughput of fully connected layers [21].
- ASICs can implement the matrix multiplication required for fully connected layers as fixed-function hardware that is optimized for both performance and power efficiency. Since ASICs are designed for specific tasks, they can achieve the highest energy efficiency for processing fully connected layers, making them ideal for mobile and embedded applications [22].

#### 2.1.5. Summary

The operations described in Section 2 are the most frequently used in convolutional neural networks. In order to accelerate these operations, one needs to understand how they work, to understand which of them represents a bottleneck. In Table 2, some of the most well-known neural networks are presented, with their number of parameters and number of required operations:

### 2.2. New Approaches to Image Processing Machine Learning Models

Newer CNN architectures for image processing tasks are highlighted in Table 3 and described in this subsection.

#### 2.2.1. Attention Mechanisms

Attention mechanisms are a more recently integrated part of most state-of-the-art deep neural networks, due to their ability to allow models to focus on relevant parts of the input data. Attention mechanisms have been integrated into convolutional neural networks (CNNs) to enhance their ability to capture intricate patterns and dependencies in images. In this section, we will take a look at the most well-known types of attention mechanisms.

##### Self-Attention

Self-attention (also known as intra-attention) is a mechanism that computes the representation of a sequence by relating different positions of the sequence to each other. In the context of CNNs, self-attention can be applied to image patches, allowing the network to capture dependencies between distant parts of the image [37].

A self-attention mechanism is typically defined by three key vectors: query *Q*, key *K*, and value *V*. The output of self-attention is computed as follows:

(22) $$Attention\left(Q,K,V\right)=softmax\left(\frac{QK^{T}}{\sqrt{d_{k}}}\right)V$$

where $Q=XW_{Q}$, $K=XW_{K}$, $V=XW_{V}$, $W_{Q}$, $W_{K}$, and $W_{V}$ are learned weight matrices; *X* is the input matrix; and $d_{k}$ is the dimensionality of the key vectors.

Self-attention mechanisms, as used in vision transformers (ViTs) [32], allow a model to consider the relationship between different parts of an image at a global scale, rather than just focusing on local features as in traditional CNNs. This global perspective is particularly beneficial for tasks like image classification, where understanding the overall structure and context of the image is crucial.

In [38], the authors highlighted the impact of attention mechanisms in various computer vision applications. For instance, in object detection, attention mechanisms help models more accurately identify and localize objects within an image by focusing on regions where objects are likely to be found. In image segmentation, attention mechanisms can improve a model’s ability to delineate objects from the background, leading to more precise segmentation maps.

##### Channel Attention

Channel attention mechanisms focus on emphasizing or suppressing different feature maps in a CNN based on their importance. This type of attention evaluates the importance of each channel (i.e., feature map) in the input tensor and scales it accordingly [39]. Channel attention can be computed as follows:

(23) $$M_{c}=\sigma \left(f_{ca}\left(AvgPool\left(X\right)\right)+f_{ca}\left(MaxPool\left(X\right)\right)\right)$$

where $M_{c}$ is the channel attention map, $f_{ca}$ represents fully connected layers followed by activation functions, AvgPool and MaxPool are global average and max pooling operations, and σ is the sigmoid function.

The effect of channel attention is that it enables the model to focus on the most informative feature maps, improving the network’s ability to capture relevant features and enhancing performance in tasks like object detection and image classification.

##### Spatial Attention

Spatial attention mechanisms focus on the spatial locations within a feature map that are most important for a given task. Unlike channel attention, which emphasizes entire feature maps, spatial attention works at the pixel level, allowing the network to focus on relevant spatial regions in the input image [40]. Spatial attention is typically calculated as follows:

(24) $$M_{s}=\sigma \left(f_{sa}\left(\left[AvgPool\left(X\right);MaxPool\left(X\right)\right]\right)\right)$$

where $M_{s}$ is the spatial attention map, $f_{sa}$ is a convolutional operation, and [;] is the channel-wise concatenation.

Spatial attention helps a network highlight important regions in an image, making it particularly useful in tasks such as segmentation and saliency detection.

##### Multi-Head Attention

Multi-head attention extends the concept of self-attention by applying several attention mechanisms in parallel (heads). Each head operates independently and captures different aspects of the input, and their outputs are concatenated and linearly transformed to form the final output [37]. The output of a multi-head attention mechanism is

(25) $$MultiHead\left(Q,K,V\right)=Concat\left(head_{1},head_{2},\ldots ,head_{h}\right)W_{O}$$

where each head is computed as

(26) $$head_{i}=Attention\left(QW_{Q_{i}},KW_{K_{i}},VW_{V_{i}}\right)$$

and $W_{Q_{i}}$, $W_{K_{i}}$, $W_{V_{i}}$ and $W_{O}$ are learned weight matrices.

Multi-head attention allows a network to attend to information from different representation subspaces, making it more robust in capturing complex dependencies in the data.

##### Co-Attention

Co-attention mechanisms are used in tasks where multiple inputs must be processed simultaneously [41], such as aligning image features with text in visual question answering (VQA). For two modalities, for example image features *F* and text features *T*, the formula can be expressed as follows:

(27) $$A_{ij}=\frac{exp(F_{i}\cdot T_{j})}{\sum_{k}exp\left(F_{i}\cdot T_{k}\right)}$$

where $A_{ij}$ is the attention weight between the *i*-th image feature and the *j*-th text feature, and $F_{i}$, $T_{j}$ are feature vectors from the image and text modalities.

##### Attention Mechanisms in Hardware Acceleration

Attention mechanisms are computationally intensive, particularly when applied to high-resolution images or large datasets. Custom hardware solutions such as GPUs, TPUs, and ASICs are crucial for accelerating these operations, making them feasible for real-time applications and large-scale deployment.

Spatial attention mechanisms, which involve computing attention maps across the spatial dimensions of an image, can be optimized on custom hardware by leveraging parallel processing capabilities. GPUs and TPUs are particularly well-suited for these operations, as they can perform the matrix multiplications and softmax operations required by attention mechanisms in parallel across multiple cores, significantly speeding up the computation.

Channel attention mechanisms, which require the computation of attention scores across feature channels, can also benefit from hardware acceleration. Custom hardware can be designed to efficiently handle the element-wise multiplications and additions involved in these operations, reducing latency and improving throughput.

Self-attention mechanisms, especially those used in vision transformers, involve computing attention scores across all pairs of pixels in an image, which can be highly computationally expensive. Custom hardware can optimize these operations by implementing dedicated units for matrix multiplication and softmax operations, which are the core components of self-attention. Additionally, hardware accelerators can exploit the inherent parallelism in self-attention to further reduce the computation time.

Multi-head attention, which involves performing multiple attention operations in parallel, is another area where custom hardware can make a significant impact. By designing hardware that can efficiently handle the parallel computation of multiple attention heads, it is possible to improve the scalability and efficiency of attention-based models, enabling them to handle larger and more complex images.

#### 2.2.2. Transformer Blocks

Transformer blocks have gained significant attention in recent years, particularly for their application in image processing tasks within convolutional neural networks (CNNs). Initially introduced for natural language processing (NLP) in the transformer model by Vaswani et al., transformer blocks have been adapted to effectively handle image data, leading to models like vision transformers (ViTs) and hybrid CNN–transformer architectures. These models leverage the power of self-attention mechanisms to capture global context, which is often challenging for traditional CNNs [32].

##### Transformer Block Structure

A typical transformer block consists of several key components:

- Multi-Head Self-Attention (MHSA): This allows the model to focus on different parts of the input image simultaneously, capturing relationships across different regions, which was previously discussed in Sections Multi-Head Attention and Self-Attention.
- Feedforward Neural Network (FFN): This is applied to each position separately and identically, usually consisting of two linear layers with ReLU activation in between.
- Layer Normalization and Residual Connections: These are critical for maintaining stable gradients and ensuring efficient training of deep networks.

Feedforward Neural Networks are defined as

(28) $$FFN\left(x\right)=max\left(0,xW_{1}+b_{1}\right)W_{2}+b_{2}$$

where $W_{1}$, $W_{2}$ are weight matrices and $b_{1}$, $b_{2}$ are bias terms.

This feedforward layer is applied identically to each position in the sequence, independently of the other positions.

##### Vision Transformers (ViTs)

Vision transformers (ViTs) represent a significant shift from traditional CNNs, by completely replacing convolutions with transformer blocks. In a ViT, an image is divided into a sequence of patches, and each patch is treated as a token in a transformer model. The self-attention mechanism allows the model to capture global information across the entire image, overcoming the local focus of traditional convolutional layers [32].

The steps necessary for a vision transformers are as follows:

- Patch Embedding: The image is split into fixed-size patches, each of which is flattened and linearly projected into the desired vector space.
- Positional Encoding: Since transformers lack the inherent inductive biases of convolutions (e.g., translation invariance), positional encodings are added to the patch embeddings to retain spatial information.

## 3. Hardware Platforms

This section reviews the hardware platforms and architectures discussed in the literature for deploying the widely used image-processing applications. The review covers both basic image processing tasks, such as filtering, and more advanced deep learning applications. We also aim to identify the common challenges faced when designing such architectures, as well as research gaps and future trends or needs.

Traditionally, the literature has used the frames-per-second (FPS) metric to assess performance and the precision metric when deep learning is involved. In addition to exploring hardware architectures that optimize FPS and precision, this work seeks to identify additional metrics for evaluating hardware architectures, particularly in the context of embedded devices, where size, cost, and power consumption are critical factors.

The selection methodology for the platforms discussed in this work primarily considered performance, power consumption, and the availability of a software stack to facilitate application deployment for developers.

The remainder of this section is organized as follows: Section 3.1 introduces relevant metrics for neural network and image processing accelerators. Section 3.2 examines traditional CPU and CPU–GPU solutions. Section 3.3 discusses techniques for designing custom accelerators using FPGAs and ASICs. Section 3.4 explores other embedded solutions, such as microcontrollers and low-power microprocessors. Section 3.5 reviews the availability of software stacks. Section 3.6 provides a comparative analysis of the platforms discussed, and Section 3.7 highlights emerging technologies that may enhance application performance.

### 3.1. Evaluation Metrics

When evaluating hardware platforms for image processing and neural network applications, developers must consider a range of performance metrics to assess the feasibility and practicality of the implementation. This subsection outlines the key metrics commonly referenced in the literature, encompassing standard metrics such as power consumption and accuracy, along with metrics more specifically tailored to particular hardware architectures.

**Qualitative metrics** refer to the subjective (based on human perception) or objective (comparison to a predefined ground truth) evaluation of the result quality or effectiveness of an image processing algorithm. These metrics are application-specific and include examples such as image processing metrics like RMSE and PSNR [42], as well as neural network inference metrics such as accuracy, mean average precision, and recall [43].

**Quantitative metrics** primarily refer to throughput (the amount of data processed per unit of time) and latency (the delay between the initiation of a command and the point at which results begin to be produced). It is important to note that, depending on the application, achieving both high throughput and low latency simultaneously may be challenging or even incompatible [44].

**Hardware-specific metrics** are performance indicators that measure hardware’s efficiency and provide insights into how well the hardware is optimized for a specific application. The following list highlights the most relevant metrics in this category:

- Power consumption and energy efficiency are two related but distinct concepts relevant for hardware architectures. While power consumption refers to the amount of electrical power that a device uses while performing a task, energy efficiency refers to how effectively a device uses power to perform a specific task, and this is often expressed as the work done per unit of energy consumed.
- Resource utilization and available resources (in the context of ASICs and FPGAs) refer to the types of hardware or logic components available in a given device and the extent to which these resources are used by a specific application. The performance of an application across different target devices can vary depending on the ratio between the available and required resources.
- The level of parallelism is relevant in image processing, where the same operations are typically repeated across multiple data points.
- Cost of ownership refers to the total cost associated with acquiring, operating, and maintaining a device.
- Reconfigurability refers to the ability of a hardware device to be reprogrammed or reconfigured after manufacturing to perform different tasks or to change its functionality.
- Development productivity refers to how efficiently engineers can design, implement, test, and deploy an application on a specific hardware target. This is directly correlated to the availability of high-level software stacks and a strong community codebase.

### 3.2. Traditional CPU–GPU Solutions

The most efficient approach for running image processing algorithms or neural network inference, considering time-to-market and accuracy, is to use a general-purpose CPU-based computer. To further accelerate both training and inference, GPUs are often integrated into these systems. The combination of multicore CPUs and highly parallel GPUs is the most widely adopted method in the literature for solving image-related tasks.

#### 3.2.1. CPU

Before delving into GPU-based systems and heterogeneous systems that rely on both a CPU and GPU for inference, it is essential first to assess the CPU-based solutions available in the literature. As we will show, evidence from the literature suggests that traditional CPU-based solutions are still preferred over deep learning GPU-based alternatives for specific applications. This is primarily because, for more straightforward tasks, GPUs tend to be I/O bound, consume more energy than CPUs, and require longer development times for applications compared to CPU-based solutions [45]. Most researchers consider a naive CPU-based implementation as the baseline when evaluating accelerated solutions on GPUs and FPGAs. This approach is often inaccurate, as acceleration techniques can also be applied to CPU-based solutions, improving their performance through algorithm-level optimizations or hardware parallelization.

Since typical image processing applications exhibit inherent parallelism [46], computations can be divided into batches down to the pixel level, with the same operations applied to each batch. Considering this, massively parallel hardware architectures are required to efficiently accelerate such applications. On the CPU side, this parallelization is achieved through processors that allow multi-threading and single instruction multiple data (SIMD) operations. Using multi-threading, the execution efficiency is increased by dividing the work into multiple tasks running on separate threads. As the name suggests, SIMD operations apply the same computation across multiple data batches, aligning perfectly with the computation model used in image processing.

Xinyao Yi performed an extensive comparative study including multithreaded and SIMD architectures [47]. In modern architectures, the number of threads can double the number of cores, due to hyper-threading technology. This boosts performance, not only by enabling parallel execution across multiple cores, but also by ensuring that each core operates near the maximum utilization. The latency introduced by operations such as memory accesses can be hidden by using more threads than the available cores. This is because, while one thread is waiting and has low CPU usage, another can utilize the same core, effectively maintaining continuous processing. Yi split multithreading methods into three categories, based on the granularity and developer workload:

- **Automated CPU multithreading** [48,49] significantly reduces the development time by automatically identifying loops suitable for parallel execution, while ensuring correct execution.
- **Explicit multithreading** using directive-based OpenMP [50] enables developers to parallelize code through compiler directives, resulting in performant, productive, and portable software [51]. The most basic directive in OpenMP is *#pragma omp parallel*, which marks parallel regions. OpenMP employs a fork–join model for parallel execution, starting with a single initial thread. This model operates in two iterative phases: when a parallel region is encountered, additional threads are spawned, and computations are distributed among them; when the parallel region ends, the additional threads are terminated. Furthermore, OpenMP offers several useful routines for managing threads, such as obtaining information about active threads, setting the number of threads for parallel execution, and determining the maximum number of threads available [52].
- **Manual multithreading** can be achieved using POSIX Threads (Pthreads) API. In this approach, the developer is responsible for implementing explicit parallelization within the application by dividing specific tasks between multiple threads. While this provides greater flexibility in how parallelization is applied, it also necessitates explicit concurrency safety mechanisms to prevent data races.

Single instruction multiple data (SIMD) architectures, as the name implies, involve a processor array capable of executing the same instructions across multiple data batches simultaneously, achieving increased performance through parallelized execution. In the past decade, both AMD and Intel, the two most prominent general-purpose CPU manufacturers, have introduced CPU architectures supporting 512-bit vector extensions: Intel’s AVX-512 [53] and ARM’s Scalable Vector Extension (SVE) [54]. Similar to multithreading approaches, SIMD-based approaches can be categorized into three types: auto-vectorization during compilation, explicit vectorization using the OpenMP SIMD directive, and manual vectorization with vector intrinsics [47].

The authors of [55] were able to boost the performance of their super-resolution algorithm implementation by a factor of 1.29 solely through algorithm-level optimizations. Additionally, Ku et al. demonstrated in [56] that specific applications, such as depth completion, can be efficiently implemented on a CPU-based platform without needing a GPU, using traditional image processing algorithms. The authors claimed that since their model is not deep-learning-based, it is robust against overfitting and runs as fast as, while performing better than, deep learning approaches, but on a CPU.

Wald et al. implemented a ray tracing application on an ×86 CPU in [57], claiming that their solution outperformed other state-of-the-art CPU and GPU implementations available at the time. However, a more recent experiment demonstrated that CPUs can only outperform GPUs for ray tracing at low resolutions [58].

With OpenMP, Aydin et al. accelerated an image segmentation application on an Intel Core i7-3630QM CPU with four cores and hyperthreading technologies [59]. Their experimental results showed that they were able to reach a speed-up factor of over 4× (compared to the naive single-core implementation) with dynamic scheduling and a chunk size of 4. This result demonstrates the previously mentioned fact that by utilizing hyper-threading, the speed-up can exceed the number of physical cores, as the cores are kept occupied for a more significant portion of the time.

Moradifar and Shahbahrami reviewed three techniques for SIMD acceleration: manual vectorization via the intrinsic programming model (IPM), explicit vectorization using OpenMP SIMD directives, and automated vectorization through compiler automation vectorization [47,60]. The highest speedup was achieved through manual vectorization using the intrinsic programming model, achieving a 52× improvement on a quad-core Intel Core i7-6700HQ compared to the base single-core implementation. The authors also provided results for varying filter sizes and image dimensions, offering a comprehensive analysis of the vectorization capabilities and potential bottlenecks.

Although the aforementioned literature provided clear evidence that there are cases where CPUs may be preferred over GPUs for specific image-processing applications, most recent research has focused on CPU–GPU systems. In these systems, the CPU is typically employed for control tasks such as scheduling and data movement or as part of heterogeneous systems, where both the CPU and GPU handle computation-intensive tasks. In our research, we identified that CPU-only systems are currently preferred for simpler applications, where the computation time is comparable to or shorter than the data transfer time, or in scenarios where GPUs are unavailable, such as in embedded systems, which are discussed in Section 3.3. A survey on deep learning training and inference performed on CPU-only applications was presented in [61]. Mittal et al. highlighted several factors that justify further research in this area, such as the high memory capacity of CPUs, high clock frequencies, and lower cost compared to other hardware solutions. They also compiled a set of techniques useful for optimizing deep neural networks for CPU-based training and inference.

#### 3.2.2. GPU

**Figure 1:**
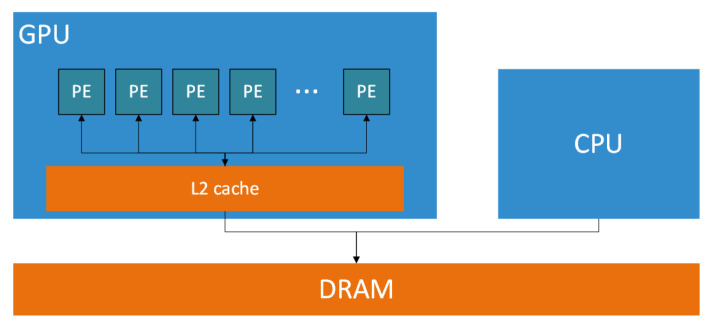
Typical GPU architecture.

Graphics processing units (GPUs) are massively parallel, special-purpose integrated circuits designed to handle a large number of simultaneous operations, making them well-suited for tasks like graphics rendering and computationally intensive workloads. From a hardware perspective, GPUs consist of many simpler processing elements (compared to CPU cores) and an L2 cache memory shared by all processing elements. They are designed to work in conjunction with a host CPU and access the host memory for optimal performance. Figure 1 presents a simplified overview of this architecture.

Nikolić et al. presented a comparison between CPU and GPU subsystem architectures and compute primitives [62]. This showed that while CPUs operate on 1 × 1 data units, featuring low latency and the ability to handle a wide variety of computational tasks, GPUs operate on 1 × N data units and are optimized for high throughput.

We believe that GPU usage has significantly increased over the past decade due to two key factors: the rising demand for parallel computing in image processing and deep learning, and the improved accessibility of GPUs, both in terms of software mechanisms that facilitate their use and their affordability. As we will demonstrate in Section 3.2, the demand for parallel computing can also be met with custom-made parallel architectures implemented in FPGAs and ASICs, in some cases with better performance compared to GPUs. This is where the second factor mentioned earlier—GPU accessibility—comes into play. GPUs are more readily available and easier to integrate, making them a practical solution for many applications compared to custom FPGA or ASIC designs. The following list details the key features of the software stacks provided by the three major GPU manufacturers, as well as a cross-platform API:

- **NVIDIA CUDA** [63], first introduced in 2006, is a comprehensive software stack optimized for NVIDIA GPUs, which accounted for approximately 77% of the discrete desktop GPU market share in 2019 [64]. It includes low-level programming capabilities for writing GPU programs, known as kernels, along with high-level libraries designed for computer vision and other specialized applications. Afif et al. conducted a study on NVIDIA CUDA [65], providing an overview of the software model, hardware architecture, and various efforts to accelerate computer vision algorithms.
- **AMD ROCm** [66] serves a similar purpose as CUDA, but is designed for AMD GPUs, including programming models tools and libraries for AI and HPC applications. A key difference between CUDA and ROCm is that the former is a closed-source platform, while the latter is an open-source software stack. Otterness et al. highlighted the advantages of an open software stack, arguing that closed-source platforms may obstruct research efforts [64]. The AMD ROCm software stack is built on the *amdkfd* driver, with several layers of APIs above it, ending in a final layer known as HIP (heterogeneous-compute interface for portability). HIP is designed to be platform-independent, allowing for seamless conversion from HIP and CUDA, and vice-versa. This provides the advantage that existing CUDA kernels can usually be converted to HIP and executed on AMD GPUs. The study by Otterness et al. offers comprehensive insights into the AMD ROCm software stack and highlights a gap in the literature and research in this domain. Their results demonstrate that, while there are instances where NVIDIA and AMD GPUs exhibit similar performance, in their tests, the AMD GPU was three times slower than its NVIDIA counterpart. The authors attributed this performance disparity to the relative immaturity of ROCm compared to the more established CUDA environment.
- **Intel oneAPI** [67] is a Data-Parallel C++ (DPC++)-based collection of tools, libraries, and frameworks designed for deployment on Intel CPUs, GPUs, and FPGAs. Intel oneAPI utilizes the SYCL [68] framework, providing an open-source, vendor-independent solution for heterogeneous computing. Alcaraz et al. performed an evaluation of Intel oneAPI in terms of usability, performance, and throughput [69]. The authors successfully implemented a heterogeneous application, deploying it on two pairs of devices using DPC++ (CPU+FPGA and CPU+integrated GPU). They concluded that, although the CPU+iGPU approach yielded the best results for image denoising, platform-specific code was still required to achieve optimal performance.
- **OpenCL** [70] is an open, royalty-free standard for cross-platform parallel programming. The primary conceptual distinction between OpenCL and oneAPI is that oneAPI is mainly designed for Intel devices, whereas OpenCL is vendor-neutral and not tied to a specific hardware manufacturer. Several studies have suggested that the generality of OpenCL often leads to performance trade-offs or even an inability to execute specific specialized applications (compared to CUDA workflow) [71,72].

#### 3.2.3. Heterogeneous Systems

In most CPU–GPU systems, the GPU serves as the primary compute unit, or accelerator, while the CPU functions as the host, responsible for data collection, pre- and post-processing, and transmitting data to the accelerator. Although the existing literature provides various definitions of heterogeneous computing [73], in this paper, we consider heterogeneous systems as those in which both the CPU and GPU act as compute units, sharing the computational load to maximize the overall utilization of their respective processing capabilities. The immediate advantage of using such systems is the potential for increased throughput by leveraging more of the available computing resources. However, this comes with the drawback of longer development times and the potential risk of increased delays due to CPU–GPU synchronization challenges.

In [52], Jang et al. accelerated a text detection neural network implementation by running the inference on a GPU and utilizing OpenMP to speed up the raw data preparation process on the CPU. By doing so, the authors achieved a 15× speedup compared to the CPU-only implementation and a 4× speedup compared to the GPU implementation without OpenMP.

Mittal et al. conducted a comprehensive review of heterogeneous computing techniques, including benchmarks for such systems. They concluded that while these systems offer the aforementioned benefits, further research is necessary to automate the compilation and deployment of code for heterogeneous environments [73].

### 3.3. Custom Accelerators

The main challenges of accelerating image processing and deep learning tasks are throughput, precision, power consumption, and area [44]. When accelerating such applications, a common trade-off arises: increasing the compute power to boost throughput and precision leads to higher power consumption and a larger area footprint. To address this challenge, a common approach is to utilize application-specific hardware accelerators, which are specifically designed as a Pareto-optimal solution that balances the trade-offs between compute power, throughput, precision, power consumption, and area. Section 3.2.1 will discuss this approach and prior research efforts related to implementing application-specific integrated circuits (ASICs) for image processing and neural networks. Section 3.2.2 will cover a similar, yet more flexible, approach involving the use of FPGAs.

#### 3.3.1. Application-Specific Integrated Circuits (ASICs)

In this section, we present various approaches found in the literature for designing custom integrated circuits (ICs) aimed at image processing and neural network deployment. Hu et al. surveyed some existing hardware accelerators for convolutional neural networks (CNNs) [74]. The authors argued that while a custom-tailored ASIC would yield optimal results in terms of throughput, energy efficiency, and size, this approach lacks scalability due to the rapid advancements and frequent changes in the field of neural networks. The authors considered the hardware-mapped neuron approach to be the traditional method, noting that modern solutions demand more sophisticated hardware, such as tensor processing units (TPU).

Machupalli et al. conducted a review of ASIC-based accelerators for neural network inference [75]. The authors categorized the existing architectures into four groups, as follows:

- **ALU-based accelerators** represent the traditional approach, featuring highly parallel, multi-core architectures composed of numerous relatively simple compute units.
- **Dataflow accelerators** are specifically designed to minimize off-chip memory operations. These architectures incorporate more complex memory hierarchies and data-movement algorithms to enhance efficiency.
- **Sparsity-based accelerators** reduce the computational and memory demands of deep neural network (DNN) inference by pruning insignificant weights and nodes in the network. While this approach lowers memory access and computational complexity, it introduces additional overhead for compressing non-zero weights and skipping zero multiplications. This necessitates more complex algorithms to ensure a performance boost.
- **Hybrid implementations**, as described by the authors, refer to the use of alternative technologies to enhance the performance of ALU-based accelerators. These technologies include analog computation, photonic computing, and quantum computing.

Boussadi et al. implemented two ASIC architectures utilizing a parallel, multi-processor approach for embedded image processing applications [76]. The first IC, referred to as HNCP-II, was implemented in 65 nm CMOS technology and contained 16 open-source processing elements. In contrast, the second IC, HNCP-III, was implemented in 28 nm FD-SOI CMOS technology and included 64 processing elements. While the area and power consumption were similar between the two implementations, HNCP-III operated at four times the frequency and had four times more processing elements. As a result, it could execute a feature detection algorithm on 1024 × 1024 inputs in 2.43 ms, compared to 38.95 ms on HNCP-II.

Di Guglielmo et al. implemented an auto-encoder ASIC for lossy data compression, synthesized in low-power 65 nm CMOS technology [77]. The use of auto-encoder-based data compression enabled the generation of multiple compression algorithms, simply by modifying the neural network weights. Since the resulting circuit was intended for use in the compact muon solenoid (CMS) experiment at the CERN Large Hadron Collider, it was implemented using a radiation-tolerant design, by incorporating triple modular redundancy (TMR) logic to ensure reliability in high-radiation environments. The expected results for a 22-bit 3 × 4 × 4 input inference on this system were 50 ns latency, 2.38 nJ/inference energy consumption, and 3.6 mm^2^ circuit area. To provide a rough estimation of the model’s complexity and configurability, the number of input parameters (weights) amounted to over 13 Kbits.

In addition to implementing complete systems for various applications, further research is required to develop new techniques and optimized building blocks specifically designed to fully exploit the energy and area efficiency potential of ASICs. Mendez et al. described in [78] the design, development, and implementation process of a power delay product (PDP) optimized computational unit targeted for medical image compression. Additionally, Thakur et al. introduced a novel, speculative parallel prefix adder for image processing applications [79]. Their implementation demonstrated performance improvements of 29% and 88% compared to existing parallel prefix adders with similar architectures. We believe that further research in the area of ASIC components is essential to identify new architectures and enhance existing ones for custom applications in image processing and neural network inference.

#### 3.3.2. Field-Programmable Gate Arrays (FPGAs)

FPGAs offer advantages that overlap with all the devices described in the previous sections: they feature low power consumption similar to ASICs, they are reconfigurable like CPUs, and they possess the massive parallelism characteristic of GPUs. Another factor that makes FPGAs highly versatile for a wide range of applications is their availability in various forms, from embedded devices to high-performance data center accelerator cards.

Considering the complexity of CPU development as a baseline, it can be stated that, due to architectural differences, GPU programming is more complex, while FPGA RTL design in Verilog or VHDL represents the most complex approach. Over the past decade, high-level synthesis (HLS) methodologies have gained significant popularity, greatly reducing the implementation time for FPGA applications. In an ideal scenario, HLS can even allow the use of the same source code as that used for CPU programming.

While traditional CPU–GPU systems primarily face challenges related to throughput and accuracy, FPGA designs encounter additional hardware implementation challenges, including resource utilization, estimated clock frequency, and energy consumption [44].

Common FPGA-based acceleration techniques include the use of parallel processing elements (SIMD approach), designing large computational circuits that ideally process and output data within a single cycle, or, given that combinational-only circuits are impractical for computation-intensive applications, implementing pipelined architectures.

Siddiqui et al. evaluated an FPGA-based soft processor for image processing called image processing processor (IPPro) [80]. Using 16 instances of IPPro, on a k-means clustering application, their results demonstrated a fps/W (frames per second per watt) performance improvement of 57, 28, and 1.7 times compared to the ARM Cortex-A7 CPU, NVIDIA GeForce GTX980 GPU, and ARM Mali-T628 embedded GPU, respectively.

DiCecco et al. presented a modified version of the Caffe Deep Learning Framework [81] in [82], incorporating additional support for FPGA deployment. To achieve FPGA deployment, the authors used the OpenCL framework. While this method enhances development productivity by adding an abstraction layer over the circuit design process, it also leads to a performance reduction. Their results showed performance drops of 2.1× and 9.4× compared to CPU and GPU implementations, respectively, and a 1.2× decrease compared to previous FPGA deployment attempts on the same target. The authors emphasized that their work serves as a proof-of-concept, demonstrating the feasibility of this approach. They suggest that further research in this area could lead to improved performance and more optimized results.

FCUDA [83] is an open-source framework that translates CUDA kernels into synthesizable C code. The C code generated by FCUDA can then be used as input for high-level synthesis (HLS) tools, to generate register transfer level (RTL) code for FPGA deployment. The literature shows several successful attempts at using FCUDA to generate different FPGA solutions based on CUDA kernels [84,85,86]. Gurmani et al. presented such an approach in [86] that combined the SIMD performance of GPU architectures with the low-power characteristics of FPGAs. The authors proposed using FCUDA [83] to translate CUDA kernels into RTL designs for deployment on FPGAs. The authors discussed the impact of synthesizing a single complex CUDA kernel versus multiple simpler kernels, arguing that the latter approach would offer more benefits, such as improved scalability, resource utilization, and ease of optimization during the synthesis process. They also mentioned that HLS-based techniques enable easy design space exploration (DSE), allowing designers to find Pareto-optimal implementations by fine-tuning the design using HLS directives. For a stereo matching application, the comparison between FPGA-based CUDA kernels and the GPU implementation in [86] showed a similar latency, but the FPGA implementation consumed 16 times less power.

### 3.4. Other Embedded/Mobile Solutions

This section aims to briefly introduce other hardware architectures used in image processing applications that have gained popularity over the past decade but do not fit into the previously mentioned categories. These devices are typically resource-constrained and have a more limited range of use cases.

**NVIDIA Jetson** [87] is a family of embedded computer targeting edge computing applications. For instance, Jetson Nano features a quad-core ARM Cortex-A57 processor and a 128-core NVIDIA CUDA GPU. It has a dedicated AI software stack and a pre-trained model zoo, enabling quick deployment of AI models. Elmanaa et al. deployed a YOLOv7-tiny model on the NVIDIA Jetson Nano platform, achieving a mean average precision (mAP) of 0.8 across four object classes, with an average inference speed of 16 FPS [88].

**USB-based accelerators**, such as the Coral USB Accelerator [89] and Intel Neural Compute Stick 2 [90], are low-power, compact co-processors designed to accelerate tensor operations. Due to their limited resources, they impose constraints on the model architectures that can be deployed, often requiring models to be adapted through quantization or layer removal.

**Microcontrollers** can provide a low-power, cost-effective edge solution for image processing and even machine learning tasks, helping to reduce data transfer latency to data centers and minimize the reliance on internet connectivity [91]. Saha et al. further extended the motivating factors of embedded AI deployment, including applicability, independence from network infrastructure, privacy, and low deployment cost [92]. Existing microcontroller-based ML software stacks either generate embedded C code that implements a model at compile time (uTensor [93], uTVM [94]), or they rely on runtime interpreters that are configured with parameters such as the model architecture and weights (e.g., TensorFlow Lite Micro [95]). Numerous attempts at running microcontroller-based applications have appeared in the literature, providing valuable starting points for new developers entering this field [91,92,96,97].

### 3.5. Software Stack

In addition to hardware acceleration, the software stack plays an important role in efficiently leveraging the computational power of specialized hardware. For example, CUDA, developed by NVIDIA, provides a comprehensive parallel computing platform and programming model tailored to NVIDIA GPUs. CUDA [63] allows developers to access the GPU’s vast resources with minimal overhead, enabling fine-grained control over parallelism and memory management, which is crucial for large-scale machine learning tasks. Similarly, TensorFlow provides optimized integration with Google’s TPUs [20], abstracting complex operations like matrix multiplications and convolutions, while ensuring that the underlying hardware can execute these operations at peak efficiency. The software ecosystem, through libraries like PyTorch, TensorFlow, and JAX, provides APIs that not only simplify the programming of high-performance hardware but also offer tools for automatic differentiation, distributed training, and model deployment across heterogeneous environments. By bridging hardware with software, these frameworks allow practitioners to focus on high-level operations without needing to manually optimize for hardware, while still taking full advantage of device-specific accelerations. This synergy between hardware and software ecosystems is crucial for the deployment of scalable, high-performance machine learning models, and warrants consideration when implementing acceleration strategies. A summary of the most popular software stacks for image processing and neural networks inference is presented in Table 4.

### 3.6. Comparison

This section compares the hardware approaches outlined above, using the metrics defined in Section 3.1: *Evaluation Metrics*. Table 5 summarizes this comparison, highlighting the best choice for each metric. The table shows that all hardware architectures, except embedded accelerators, excel in at least one specific metric relative to the others, also indicating that no single solution is universally optimal. This occurs because there is typically a trade-off between these metrics, often with a direct negative correlation between them. Embedded accelerators are a unique category of hardware designed to provide specialized acceleration within constrained environments, such as edge devices, IoT devices, and portable applications. While they may not achieve peak performance, reconfigurability, or low power consumption compared to dedicated hardware like ASICs or high-end GPUs, embedded accelerators are valuable because they offer a balance of these metrics within limited size, power, and resource budgets.

It is worth mentioning that our work does not focus on reviewing specific implementations, but rather on examining the range of generic platforms available. Furthermore, given the broad diversity of platforms—from high-performance heterogeneous CPU–GPU systems to low-cost, low-power microcontrollers—a comparison would reveal substantial performance disparities. In Section 4, *Discussions*, we address this topic, emphasizing that while performance and throughput are significant, they are not always the primary metrics. Application engineers should seek the fastest, most efficient solution, while also accounting for the particular constraints of their use case.

Although all platforms discussed in this work are suitable for real-time applications, it is essential to examine two edge-case scenarios: resource-limited devices, like microcontrollers, and high-performance, high-accuracy devices, such as high-end GPUs. Cloud computing presents an effective solution for combining these devices in a collaborative setup. In this approach, low-power, cost-effective devices deployed at the edge can collect data or perform initial processing with minimal energy consumption. By streaming data from edge microcontrollers to the cloud, the system can offload computationally intensive tasks, such as inference and image processing, to high-performance cloud servers. FPGAs hold a unique position, as they come in various configurations that allow them to function as either edge nodes or datacenter acceleration cards. This flexibility enables them to scale across the entire spectrum, from affordable, low-profile devices to high-performance, power-intensive units.

### 3.7. New Emerging Technologies

Emerging technologies like neuromorphic computing and quantum accelerators offer promising directions for the future of image processing, particularly in fields requiring vast computational resources and novel architectures. Neuromorphic computing, inspired by the human brain, uses spiking neural networks (SNNs) and custom hardware such as Intel’s Loihi to process information more efficiently, particularly for real-time image recognition tasks [98]. This approach mimics the brain’s energy-efficient spike-based communication and could potentially revolutionize low-power image processing applications, such as edge computing in IoT devices [99]. On the other hand, quantum accelerators, leveraging qubits and quantum entanglement, present opportunities for solving complex optimization problems faster than classical computing methods. While using quantum computing in image processing is still in its infancy, early research has shown potential for accelerating high-dimensional image classification tasks and optimizing machine learning models [100]. Though not yet widely adopted, these emerging technologies could complement traditional hardware accelerators by addressing power consumption challenges and enabling new paradigms for processing large-scale, high-resolution images. Optical computing is an area where light, rather than electricity, performs computations. Optical computing promises to accelerate data processing by several orders of magnitude while reducing power consumption, thanks to the speed of light and the parallelism inherent in optical systems. This technology is particularly beneficial for large-scale image processing tasks where traditional silicon-based processors struggle to keep up [101]. Another emerging field is DNA computing, which uses biological molecules like DNA to perform complex calculations. DNA computing leverages the massive parallelism inherent in molecular interactions, allowing the processing of vast datasets in a highly parallel and energy-efficient manner. While still in its early stages, DNA computing could revolutionize areas that require high throughput, such as cryptography and large-scale pattern recognition [102]. Spintronics is an advancing technology that exploits the spin of electrons, in addition to their charge, for information processing. Spintronic devices have the potential to create memory and logic circuits that are faster and consume less energy than current semiconductor technologies, offering new possibilities for real-time image processing and edge computing [103].

Table 6 summarizes these technologies, together with the associated advantages, disadvantages, and the hardware platforms they are suited for.

## 4. Discussions

Thus far, our paper has not emphasized key aspects such as cost of ownership, power consumption, and precision, as these factors are highly dependent on the specific target device and application. Moreover, previous research articles often failed to provide a comprehensive evaluation across all relevant performance metrics, focusing instead on a limited subset that may not fully capture the needs of modern applications. In this section, we aim to address these metrics and encourage future research to adopt a broader, more complete analysis that considers the full spectrum of performance factors.

We believe many research efforts are unfairly overlooked due to poor results in a single metric, which is a narrow and misguided perspective. One of the primary goals of deep learning and image processing algorithms is to continuously enhance the accuracy with each new generation. While high accuracy is vital for critical applications such as medical imaging or search and rescue, we argue that in some contexts, accuracy can be traded for other factors, such as real-time processing capabilities and low cost of ownership. For example, edge devices for wildlife monitoring, recommendation systems, or content filtering may tolerate occasional misclassifications without significant consequences.

We encourage future research to (1) develop a more comprehensive set of evaluation metrics that reflect the diversity of applications in neural networks and image processing hardware, and (2) consistently apply these metrics in their assessments. There will always be trade-offs among factors like performance, power consumption, and precision, and the Pareto-optimal solution may not always be the best choice. In some cases, a less optimal implementation might be the only viable option, due to hardware constraints, making it more appropriate for certain specific use cases.

## 5. Conclusions

The deployment of neural networks in image processing faces significant challenges related to computational complexity, memory constraints, and energy efficiency. Key operations such as convolutions, pooling, and non-linear activations demand specialized hardware accelerators, including GPUs, TPUs, and FPGAs. Current hardware platforms still struggle with optimizing real-time performance and scaling for large models. Research gaps remain in efficient model compression, quantization techniques, and adaptability across heterogeneous platforms. Bridging these gaps is crucial for advancing both hardware and algorithmic co-design in the evolving landscape of deep learning applications.

## 6. Future Directions

Through our research, we identified the following gaps, which can be further explored as potential directions for future studies:

- Most general-purpose FPGA-based accelerators for image processing and neural network inference lack comprehensive software stacks for easy model deployment, especially compared to the well-established ecosystems for CPUs and GPUs (e.g., TensorFlow, PyTorch). Future research could explore three possible directions: (1) integrating support for new accelerators within existing software stacks, (2) providing each new accelerator with a high-level software API, or (3) developing a unified software methodology that supports deployment across various hardware platforms.
- Further research on low-end devices is necessary, as there is still significant potential for performance improvements. Additionally, as previously mentioned, there is a clear market demand for such devices.
- New metrics should be developed and their adoption promoted, to provide deeper insights into the capabilities and potential use cases of each newly developed accelerator.

## Figures and Tables

**Table 1:** Usual operations performed in convolutional neural networks.

	AlexNet [1]	VGG16 [2]	GoogleNet ^1^ [3]	ResNet ^1^ [4]	DenseNet [5]	MobileNet [6]	EfficientNet [7]
Convolution	X	X	X	X	X	X	X
Pooling	X	X	X	X	X	X	X
Activation	X	X	X	X	X	X	X
Regularization	X	N/A	X	X	N/A	X	N/A
FC	X	X	X	X	X	X	X

^1^ Some network-specific operations not included in this table can still be replicated using the listed operations.

**Table 2:** State-of-the-art CNN models and their operations [30].

Model	Input Size	Param. Size (MB)	Operations (GOPs)
AlexNet	227 × 227	233	0.73
Squeezenet	224 × 224	5	0.84
VGG-16	224 × 224	528	16
VGG-19	224 × 224	548	20
GoogleNet	224 × 224	51	2
Resnet-18	224 × 224	45	2
Resnet-152	224 × 224	230	11
Inception-V3	299 × 299	91	6
Densenet-201	224 × 224	77	4
MCN-mobileNet	224 × 224	16	0.58

**Table 3:** State-of-the-art CNN models and their operations.

	EfficientNet V2 [31]	Vision Transformer (ViT) [32]	Swin Transformer [33]	ConvNeXt [34]	CoAtNet [35]	CaiT (Class-Attention in Image Transformers) [36]
Fused MBConv Blocks	X	-	-	-	-	-
Swish	X	-	-	-	X	-
Squeeze-and-Excitation	X	-	-	-	-	-
Transformer Blocks	-	X	X	-	-	X
Attention	-	X	-	-	X	X
Layer Normalization	-	X	X	X	-	X
Depthwise Separable Convolutions	-	-	-	X	-	-

**Table 4:** Comparison of software stacks for hardware acceleration.

Software Stack	Supported Hardware	Parallelism Support	Key Features	Limitations
CUDA	NVIDIA GPUs	Explicit parallelism, memory management	Custom kernel support, direct GPU control	Limited to NVIDIA hardware
TensorFlow	NVIDIA GPUs, TPUs, CPUs	Automatic parallelism with user options	High-level API, TPU integration, auto differentiation	Not optimized for AMD GPUs
PyTorch	NVIDIA GPUs, TPUs, CPUs	Automatic parallelism with custom control	Flexible API, dynamic graph building, distributed training	Requires custom implementation for specific hardware
JAX	NVIDIA GPUs, TPUs, CPUs	Automatic parallelism with vectorization and compilation	XLA compiler support, functional programming	Limited to backends supported by XLA
OpenCL	Multi-platform (CPUs, GPUs, FPGAs)	Explicit parallelism, multi-platform support	Cross-platform, open standard	Requires manual optimization for performance
Rocm	AMD GPUs	Explicit parallelism for AMD GPUs	AMD GPU optimization, HIP support	Limited hardware support outside AMD

**Table 5:** Comparison of different hardware architectures, with highlighted entries indicating the best choice for each metric.

	Power Consumption	Cost of Ownership	Reconfigurability	Productivity	Performance
GP-CPU	Moderate to high	Moderate	Moderate	**High**	Moderate
GP-GPU	High	Moderate to high	Moderate	**High**	High
ASIC	**Optimized for performance**	High	None	Low	Very high
FPGA	Low to moderate	Moderate to high	**High**	Moderate	Moderate to high
Embedded accelerators	Low to moderate	Low to moderate	Low to moderate	Moderate to high	Moderate
Microcontrollers	**Very low**	**Low**	Low	Low to moderate	Low to moderate

**Table 6:** Comparison of emerging technologies for image processing.

Technology	Advantages	Disadvantages	Suited Hardware Platforms
Neuromorphic Computing	Low-power, brain-inspired, real-time processing, energy efficiency	Limited software ecosystem, still maturing, not suitable for general-purpose tasks	Specialized neuromorphic chips (e.g., Intel Loihi)
Quantum Computing	Solves complex optimization problems, high parallelism, potential for massive speedup	Still in early stages, high error rates, very sensitive to noise, requires cryogenic temperatures	Quantum processors (e.g., D-Wave, IBM Q, Google Sycamore)
Optical Computing	High-speed data processing, reduced power consumption, intrinsic parallelism with light	Technological immaturity, limited adoption, costly, complex light-based infrastructure	Optical circuits and photonic processors
DNA Computing	Massive parallelism, low energy use, high-density data storage and computation	Extremely early-stage technology, complex biological interactions, hard to scale	Biological and synthetic molecular systems
Spintronics	Energy-efficient, faster data processing using electron spin, scalable for next-gen computing	Still in development, requires new infrastructure, complex physics, limited commercial adoption	Spintronic devices, future generation computing hardware

## Data Availability

No new data were created or analyzed in this study.

## Abbreviations

The following abbreviations are used in this manuscript:

GP-CPU	General Purpose Central Processing Unit
GP-GPU	General Purpose Graphics Processing Unit
CNN	Convolutional Neural Network
GPU	Graphics Processing Unit
TPU	Tensor Processing Unit
FPGA	Field Programmable Gate Array
ASIC	Application-Specific Integrated Circuit
CPU	Central Processing Unit
SIMD	Single Instruction Multiple Data
ReLU	Rectified Linear Unit
ELU	Exponential Linear Unit
FC	Fully Connected
VQA	Visual Question Answering
NLP	Natural Language Processing
MHSA	Multi-Head Self-Attention
FFN	Feedforward Neural Network
ViT	Vision Transformers
SNN	Spike Neural Network
POSIX	Portable Operating System Interface
API	Application Programming Interface
AMD	Advanced Micro Devices
SVE	Scalable Vector Extension
IPM	Intrinsic Programming Model
HPC	High-Performance Computer
HIP	Heterogeneous-Compute Interface for Portability
ALU	Arithmetic-Logical Unit
DNN	Deep Neural Network
CMOS	Complementary Metal Oxide Semiconductor
CMS	Compact Muon Solenoid
TMR	Triple Modular Redundancy
PDP	Power Delay Product
HLS	High Level Synthesis
RTL	Register Transfer Level
DSE	Design Space Exploration
